# A conserved fold for fimbrial components revealed by the crystal structure of a putative fimbrial assembly protein (BT1062) from *Bacteroides thetaiotaomicron* at 2.2 Å resolution

**DOI:** 10.1107/S1744309110006548

**Published:** 2010-07-06

**Authors:** Qingping Xu, Polat Abdubek, Tamara Astakhova, Herbert L. Axelrod, Constantina Bakolitsa, Xiaohui Cai, Dennis Carlton, Connie Chen, Hsiu-Ju Chiu, Michelle Chiu, Thomas Clayton, Debanu Das, Marc C. Deller, Lian Duan, Kyle Ellrott, Carol L. Farr, Julie Feuerhelm, Joanna C. Grant, Anna Grzechnik, Gye Won Han, Lukasz Jaroszewski, Kevin K. Jin, Heath E. Klock, Mark W. Knuth, Piotr Kozbial, S. Sri Krishna, Abhinav Kumar, David Marciano, Daniel McMullan, Mitchell D. Miller, Andrew T. Morse, Edward Nigoghossian, Amanda Nopakun, Linda Okach, Christina Puckett, Ron Reyes, Natasha Sefcovic, Henry J. Tien, Christine B. Trame, Henry van den Bedem, Dana Weekes, Tiffany Wooten, Andrew Yeh, Jiadong Zhou, Keith O. Hodgson, John Wooley, Marc-Andre Elsliger, Ashley M. Deacon, Adam Godzik, Scott A. Lesley, Ian A. Wilson

**Affiliations:** a Joint Center for Structural Genomics, http://www.jcsg.org, USA; b Stanford Synchrotron Radiation Lightsource, SLAC National Accelerator Laboratory, Menlo Park, CA, USA; c Protein Sciences Department, Genomics Institute of the Novartis Research Foundation, San Diego, CA, USA; d Center for Research in Biological Systems, University of California, San Diego, La Jolla, CA, USA; e Program on Bioinformatics and Systems Biology, Burnham Institute for Medical Research, La Jolla, CA, USA; f University of California, San Diego, La Jolla, CA, USA; g Department of Molecular Biology, The Scripps Research Institute, La Jolla, CA, USA; h Photon Science, SLAC National Accelerator Laboratory, Menlo Park, CA, USA

**Keywords:** DUF1812, PF08842, pili, fimbriae, BT1062, Mfa2, PGN0288, PG0179

## Abstract

BT1062 from *Bacteroides thetaiotaomicron* is a homolog of Mfa2 (PGN0288 or PG0179), which is a component of the minor fimbriae in *Porphyromonas gingivalis*. The crystal structure of BT1062 revealed a conserved fold that is widely adopted by fimbrial components.

## Abstract 2

The crystal structure of BT1062 from *Bacteroides thetaiotaomicron* revealed a conserved fold that is widely adopted by fimbrial components.

## 1. Introduction

The Gram-negative anaerobic bacterium *Bacteroides thetaiotaomicron* is a predominant member of the mammalian intestinal microbiota. It is important for the study of the symbiotic relationship between bacteria and humans, as well as for its abilities to digest complex plant polysaccharides and host-derived polysaccharides (Xu *et al.*, 2003 ▶). It is also an opportunistic pathogen and can cause serious infections. Extracellular proteins are expected to be crucial for such functions in *B. thetaiotaomicron* and other gut microbes. Therefore, we initiated a project to characterize the structures of proteins that are specific to the gut environment from the bacterial secretome of human gut microbiota, in order to gain further insights into the molecular mechanisms of bacteria–host symbiosis as well as of bacterial pathogenesis.

Here, we report the 2.2 Å crystal structure of a putative fimbrial assembly protein BT1062 from *B. thetaiotaomicron*, which was determined using the high-throughput pipeline of the Joint Center for Structural Genomics (JCSG; Lesley *et al.*, 2002 ▶) as part of the National Institute of General Medical Sciences Protein Structure Initiative (PSI; http://www.nigms.nih.gov/Initiatives/PSI/). The *BT1062* gene of *B. thetaiotaomicron* encodes a predicted lipoprotein with a molecular weight of 36 535 Da (residues 1–317) and a calculated isoelectric point of 4.8.

## 2. Materials and methods

### 2.1. Protein production and crystallization

Clones were generated using the Polymerase Incomplete Primer Extension (PIPE) cloning method (Klock *et al.*, 2008 ▶). The gene encoding BT1062 (Swiss-Prot Q8A8V5) was amplified by polymerase chain reaction (PCR) from *B. thetaiotaomicron* VPI-5482 genomic DNA using *PfuTurbo* DNA polymerase (Stratagene) and I-PIPE (Insert) primers (forward primer 5′-ctgtacttccagggcGCTTCATGCG­ACAGCTTTAATGAAGACC-3′, reverse primer 5′-aattaagtcgcgtta­TTGATTCTCTTCCTGAATGCGATGCACC-3′; target sequence in upper case) that included sequences for the predicted 5′ and 3′ ends. The expression vector pSpeedET, which encodes an amino-terminal tobacco etch virus (TEV) protease-cleavable expression and purification tag (MGSDKIHHHHHHENLYFQ/G), was PCR-amplified with V-PIPE (Vector) primers (forward primer 5′-taacgcgacttaatta­actcgtttaaacggtctccagc-3′, reverse primer 5′-gccctggaagtacaggttttcgt­gatgatgatgatgatg-3′). V-PIPE and I-PIPE PCR products were mixed to anneal the amplified DNA fragments together. *Escherichia coli* GeneHogs (Invitrogen) competent cells were transformed with the I-­PIPE/V-PIPE mixture and dispensed onto selective LB–agar plates. The cloning junctions were confirmed by DNA sequencing. Using the PIPE method, the gene segment encoding residues Met1–Glu22 was deleted as it was predicted to code for a signal peptide at the start of the protein. Expression was performed in a selenomethionine-containing medium at 310 K with suppression of normal methionine synthesis. At the end of fermentation, lysozyme was added to the culture to a final concentration of 250 µg ml^−1^ and the cells were harvested and frozen. After one freeze–thaw cycle, the cells were sonicated in lysis buffer [50 m*M* HEPES pH 8.0, 50 m*M* NaCl, 10 m*M* imidazole, 1 m*M* tris(2-carboxyethyl)phosphine–HCl (TCEP)] and the lysate was clarified by centrifugation at 32 500*g* for 30 min. The soluble fraction was passed over nickel-chelating resin (GE Healthcare) pre-equilibrated with lysis buffer, the resin was washed with wash buffer [50 m*M* HEPES pH 8.0, 300 m*M* NaCl, 40 m*M* imidazole, 10%(*v*/*v*) glycerol, 1 m*M* TCEP] and the protein was eluted with elution buffer [20 m*M* HEPES pH 8.0, 300 m*M* imidazole, 10%(*v*/*v*) glycerol, 1 m*M* TCEP]. The eluate was buffer-exchanged with TEV buffer (20 m*M* HEPES pH 8.0, 200 m*M* NaCl, 40 m*M* imidazole, 1 m*M* TCEP) using a PD-10 column (GE Healthcare) and incubated with 1 mg TEV protease per 15 mg of eluted protein. The protease-treated eluate was run over nickel-chelating resin (GE Healthcare) pre-equilibrated with HEPES crystallization buffer (20 m*M* HEPES pH 8.0, 200 m*M* NaCl, 40 m*M* imidazole, 1 m*M* TCEP) and the resin was washed with the same buffer. The flowthrough and wash fractions were combined and concentrated to 19.1 mg ml^−1^ by centrifugal ultrafiltration (Millipore) for crystallization trials. BT1062 was crystallized by mixing 100 nl protein solution with 100 nl crystallization solution above a 50 µl reservoir volume using the nanodroplet vapor-diffusion method (Santarsiero *et al.*, 2002 ▶) with standard JCSG crystallization protocols (Lesley *et al.*, 2002 ▶). The crystallization reagent consisted of 1.4 *M* sodium citrate, 0.1 *M* HEPES pH 7.5. A cube-shaped crystal of approximate dimensions 40 × 40 × 30 µm was harvested after 23 d at 277 K for data collection. Ethylene glycol was added to the crystal as a cryoprotectant to a final concentration of 10%(*v*/*v*). Initial screening for diffraction was carried out using the Stanford Automated Mounting (SAM) system (Cohen *et al.*, 2002 ▶) and an X-ray microsource at Stanford Synchrotron Radiation Lightsource (SSRL, Menlo Park, California, USA).

The oligomeric state of BT1062 in solution was determined using a 1 × 30 cm Superdex 200 column (GE Healthcare) coupled with miniDAWN static light-scattering (SEC/SLS) and Optilab differential refractive-index detectors (Wyatt Technology). The mobile phase consisted of 20 m*M* Tris–HCl pH 8.0, 150 m*M* NaCl and 0.02%(*w*/*v*) sodium azide. The molecular weight was calculated using *ASTRA* v.5.1.5 software (Wyatt Technology).

### 2.2. Data collection, structure solution and refinement

Multi-wavelength anomalous diffraction (MAD) data were collected on beamline 9-2 at the SSRL at wavelengths corresponding to the inflection (λ_1_), high-energy remote (λ_2_) and peak (λ_3_) of a selenium MAD experiment. The data sets were collected at 100 K using an MAR CCD 325 detector. The MAD data were integrated and reduced using *MOSFLM* and scaled with *SCALA*. Selenium sites were located using *SHELXD* (Sheldrick, 2008 ▶) and refined using *autoSHARP* (mean figure of merit of 0.46 with ten selenium sites; Bricogne *et al.*, 2003 ▶). Phase refinement and automatic model building were performed with *RESOLVE* (Terwilliger, 2003 ▶). Model completion and refinement were performed with *Coot* (Emsley & Cowtan, 2004 ▶) and *REFMAC* (Winn *et al.*, 2003 ▶). The refinement included experimental phase restraints in the form of Hendrickson–Lattman coefficients and TLS refinement with one TLS group per chain. *CCP*4 programs were used for data conversion and other calculations (Collaborative Computational Project, Number 4, 1994 ▶). Data-processing and refinement statistics are summarized in Table 1 ▶.

### 2.3. Validation, deposition and figures

The quality of the crystal structure was analyzed using the JCSG Quality Control server, which verifies the stereochemical quality of the model using *AutoDepInputTool* (Yang *et al.*, 2004 ▶), *MolProbity* (Lovell *et al.*, 2003 ▶) and *WHAT IF* v.5.0 (Vriend, 1990 ▶), the agreement between the atomic model and the data using *SFCHECK* v.4.0 (Collaborative Computational Project, Number 4, 1994 ▶) and *RESOLVE* (Terwilliger, 2003 ▶), the protein sequence using *ClustalW* (Thompson *et al.*, 1994 ▶), the atomic occupancies using *MOLEMAN*2 (Kleywegt, 2000 ▶) and the consistency of NCS pairs. It also evaluates the differences in *R*_cryst_/*R*_free_, expected *R*_free_/*R*_cryst_ and maximum/minimum *B* values by parsing the refinement log file and PDB header. All molecular graphics were prepared with *PyMOL* (DeLano Scientific). Sequence alignments were rendered using *TEXshade* (Beitz, 2000 ▶).

## 3. Results and discussion

### 3.1. Sequence analysis and functional assignment

**Figure 1:**
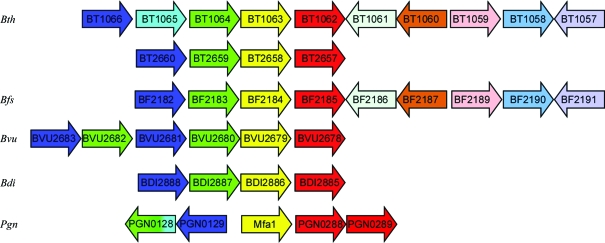
Gene context for representative BT1062 homologs in *B. thetaiotaomicron* (*Bth*), *B. fragilis* NCTC 9343 (*Bfs*), *B. vulgatus* ATCC 8482 (*Bvu*), *Parabacteroides distasonis* ATCC 8503 (*Bdi*) and *Porphyromonas gingivalis* strain ATCC 33277 (*Pgn*). The lengths of the genes are not drawn to scale. Each homologous set of sequences is represented by one color.

BT1062 is a member of a functionally uncharacterized protein family [Pfam PF08842 or DUF1812 (domain of unknown function family 1812)] consisting of ∼80 *Bacteroidetes* proteins of around 300–400 residues. Homologous proteins are abundant in *Bacteroidetes* genomes. For example, at least four paralogs are found in *B. theta­iotaomicron* VPI-5482 (BT1062, BT2657, BT4225 and BT4226; sequence identity of >20%), three in *Porphyromonas gingivalis* (PGN0185, PGN0288 and PGN0289) and eight in *B. fragilis* NCTC 9343 (BF1578, BF1851, BF1976, BF2185, BF2264, BF2871, BF3328 and BF4229). The genomic context is conserved for *BT1062* homologs, which involves a cluster of four associated genes: *BT1066*, *BT1065*, *BT1063* and *BT1062* (Fig. 1 ▶). *BT1062* and *BF2185* of *B. fragilis* have almost identical genomic environments. A cluster of genes *BT1062–BT1068*, which are likely to be an operon, all contain signal peptides and are predicted to encode lipoproteins (with the exception of BT1064). This putative operon may be under the control of *BT1069*, which encodes a putative transcription regulator. Downstream of the operon is a putative histidine kinase (*BT1058*).

**Figure 2:**
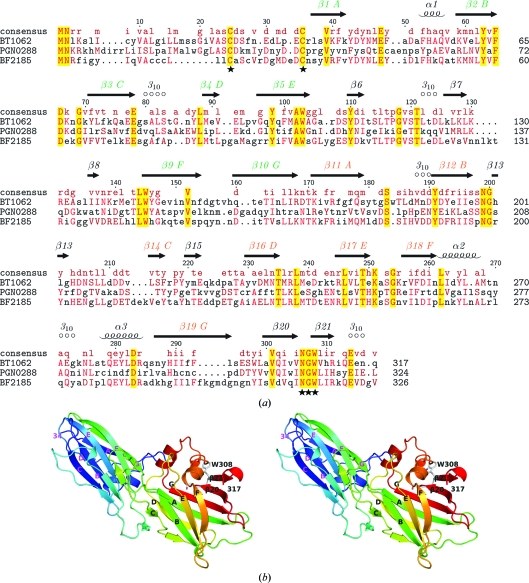
Crystal structure of BT1062. (*a*) Sequence alignment of BT1062, BF2185 and PGN0288 (Mfa2). The secondary-structural elements, residue numbering of BT1062 and consensus are shown at the top. The seven conserved β-strands (*A*–*G*) of the two transthyretin-like domains are highlighted. The potential membrane-attachment site and Mfa1-interaction site are labeled by stars at the bottom. (*b*) Stereo ribbon diagram of BT1062 monomer color coded from the N-terminus (blue) to the C-terminus (red).

BT1062 is homologous to Mfa2 (PGN0288, also previously known as PG0179) of *P. gingivalis* strain ATCC 33277 (19% sequence identity; Fig. 2 ▶ *a*). *Mfa*2 co-transcribes with the minor fimbrial antigen (*mfa*1) and is involved in the assembly of Mfa1 fimbriae (Chung *et al.*, 2000 ▶; Hasegawa *et al.*, 2009 ▶). BT1063 is a remote homolog of Mfa1 (PGN0287; 15% identity; Fig. 1 ▶), which is the structural subunit of *P. gingivalis* minor fimbriae (Yoshimura *et al.*, 2009 ▶). *P. gingivalis* also contains homologous proteins to BT1064 and BT1066 (PGN0128 and PGN0179; PGN0129 and PGN0178). BT1065 matches the N-terminal domain of PGN0128, indicating that PGN0128 is a fusion product of BT1065-like and BT1064-like proteins. Therefore, the *BT1062–BT1068* genes are most likely to encode a fimbriae (or pili) system similar to that of the minor fimbriae of *P. gingivalis*, with *BT1062* being equivalent to *mfa2*. *P. gingivalis* has at least two types of fimbriae: major (long) fimbriae with FimA as the main structural subunit (Yoshimura *et al.*, 1984 ▶) and minor (short) Mfa1 fimbriae (Hamada *et al.*, 1996 ▶; Park *et al.*, 2005 ▶). Fimbriae were also observed in strains of *B. thetaiotaomicron* and *B. fragilis* (Shinjo & Kiyoyama, 1984 ▶); however, the fimbriae-assembly machinery are currently uncharacterized at the molecular level. The similarity of the potential fimbriae proteins to those of *P. gingivalis* could suggest a similar fimbriae-assembly system in *B. thetaiotaomicron* and *B. fragilis*. The fimbriae in gut bacteria, such as *B. thetaiotaomicron* and *B. fragilis*, may be required for adhesion to host tissues (Pumbwe *et al.*, 2006 ▶), formation of biofilms with other bacteria in the gut, or play other as yet unknown functional roles.

### 3.2. Overall structure

The selenomethionine derivative of BT1062 (residues 23–317) with an N-terminal His tag was expressed in *E. coli* and purified by metal-affinity chromatography. The predicted N-terminal signal peptide (residues 1–22) was not included in the construct. The crystal structure of BT1062 was determined in the tetragonal space group *P*4_1_2_1_2 at 2.2 Å resolution using the MAD method. The final BT1062 model includes a monomer (residues 34–317; Fig. 2 ▶ *b*), one ethylene glycol and 174 water molecules in the asymmetric unit. The Matthews co­efficient (*V*_M_; Matthews, 1968 ▶) for BT1062 is 3.25 Å^3^ Da^−1^ and the estimated solvent content is 62%. The Ramachandran plot produced by *MolProbity* shows that 96.8 and 100% of the residues are in the favored and allowed regions, respectively. BT1062 is composed of 21 β-strands (β1–β21), three α-helices (α1–α3) and five 3_10_-helices. The total β-sheet, α-helical and 3_10_-helical contents are 43.0, 6 and 5.3%, respectively. BT1062 is likely to exist as a monomer in solution, which is consistent with crystal-packing analysis and analytical size-exclusion chromatography.

### 3.3. Structural comparisons

**Figure 3:**
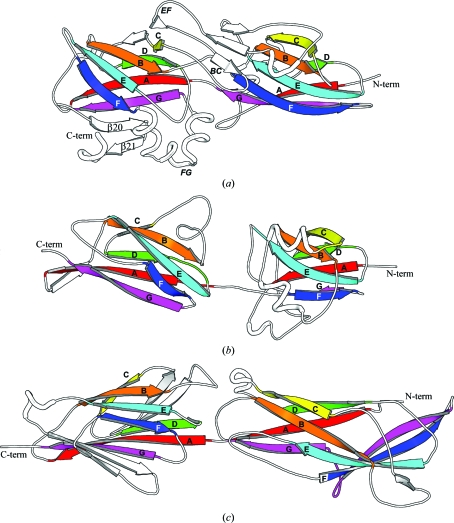
Structural comparisons of (*a*) BT1062, (*b*) the minor pilin of GBS52 (PDB code 2pz4) and (*c*) the major pilin Spy0128 (PDB code 3b2m). All molecules are shown in a similar orientation with the same scale. The conserved core strands are labeled from *A* to *G*.

The structure of BT1062 consists of a tandem repeat of two domains: I (34–170) and II (171–317). Using individual domains, the *DALI* structural similarity search server (Holm & Sander, 1995 ▶)  indicated that both domains have transthyretin-like (previously known as prealbumin-like) folds with seven core β-strands (*A*–*G*) arranged in two sheets (*DAG* and *CBEF*; Fig. 2 ▶). The top hit for domain I is the C-terminal transthyretin subdomain of the carboxy­peptidase D domain II (Aloy *et al.*, 2001 ▶; PDB code 1h8l; *Z* = 7.0, r.m.s.d. of 2.1 Å for 78 aligned C^α^ atoms, 13% sequence identity). The best match for domain II is human transthyretin (Karlsson & Sauer-Eriksson, 2007 ▶; PDB code 2qel; *Z* = 6.1, r.m.s.d. of 3.6 Å for 96 aligned C^α^ atoms, 7% sequence identity). For the entire structure, the minor pilin GBS52 of the Gram-positive bacterium *Streptococcus agalactiae* (Krishnan *et al.*, 2007 ▶) is among the top hits (fifth), with an r.m.s.d. of 5.7 Å for 145 aligned C^α^ atoms (PDB code 2pz4; *Z* = 4.1, 10% sequence identity). *TM-align* (Zhang & Skolnick, 2005 ▶) aligned BT1062 to GBS52 with an r.m.s.d. of 4.8 Å for 175 C^α^ atoms. Despite the large r.m.s.d. value, this match is significant since both proteins are fimbrial components. The two domains of both proteins have an identical fold (*i.e.* the same topology of the seven core strands). GBS52 does not have long inserts between core β-strands in its two domains, except for the *BC* loop of the first domain, while BT1062 contains several significant insertions between core strands in both domains (Fig. 2 ▶). The most significant additional structural feature of BT1062 is a small β-sheet at the domain boundary formed by the *EF* loop of domain I and the *BC* loop of domain II (Fig. 3 ▶). Domain II contains a three-helix insertion between strands *F* and *G* as well as a β-hairpin attachment (β20–β21) at the C-terminus. Thus, domain II of BT1062 deviates more significantly from the prototypical seven-stranded core domain, although some members of the transthyretin family have an additional β-strand that would correspond to β20. A similar two-domain arrangement is also observed for the *S. pyogenes* major pilin Spy0128 (*TM-align* r.m.s.d. of 5.3 Å for 178 aligned C^α^ atoms; Kang *et al.*, 2007 ▶; Fig. 3 ▶). Given the overall structural similarity and functional overlap, it seems possible that these pilin components might be derived from a common ancestral fold through divergent evolution. The basic fold of the seven core strands in these proteins has previously been described as IgG-like (Krishnan *et al.*, 2007 ▶). We have avoided such a description here owing to a lack of clear evidence to establish an evolutionary relationship between the IgG-like fold (SCOP ID 48725) and the transthyretin-like fold (SCOP ID 49451) (Andreeva *et al.*, 2004 ▶).

### 3.4. A conserved fold for fimbrial components

A sequence-similarity search using *PSI-BLAST* against the non­redundant (nr) database at the National Center for Biotechnology Information (NCBI) indicated that the family size of DUF1812 can be significantly expanded, with >1000 hits almost exclusively from *Bacteroidetes*. There are 35 potential homologs from *B. thetaiota­omicron* alone, indicating the popularity of this fold in this bacterium. Interestingly, the identified homologs include components of both the major fimbriae and the minor fimbriae of *P. gingivalis*. In addition to the BT1062 homologs in minor fimbriae discussed above, major fimbrial components, such as FimA and the accessory proteins FimC, FimD and FimE, are expected to adopt a similar fold to BT1062. Thus, DUF1812 is a collection of diverse proteins that are likely to be fimbrial components. These proteins are likely to be adapted from a single fold to serve different functions. Many of these remote homologs also contain the highly conserved tryptophan (Trp308 in BT1062) described above.

The details of the biogenesis of Mfa1-like fimbriae are still unclear. A recent study suggested that *P. gingivalis* Mfa2 is likely to anchor the Mfa1 fimbriae to the outer membrane and to regulate the length of the Mfa1 filament (Hasegawa *et al.*, 2009 ▶). Mfa2 is present in the outer membrane and may directly interact with Mfa1. Most sequence homologs of BT1062 and Mfa2 contain two highly conserved cysteines at the N-terminus (Cys25 and Cys35) located near the tip of the bilobal molecule. The first invariant cysteine was predicted to be the lipoprotein signal-peptide cleavage site (between 24 and 25) by the *LipoP* server (Juncker *et al.*, 2003 ▶). This cysteine is likely to be the last residue of the lipoprotein signal-sequence motif [lipobox motif (L/V)*XX*C, *X* = A/S/G/T] and is directly involved in membrane attachment of the matured lipoprotein *via* a thioether bond (Braun & Wu, 1994 ▶). The role of the second conserved cysteine is currently not clear. It may also be involved in membrane attachment owing to its close proximity to the first cysteine. The conformation of the peptide between the two conserved cysteines is likely to be flexible since residues 24–33 are exposed to solvent and disordered in the crystal with no interpretable electron density. The most conserved surface residues of BT1062 homologs correspond to a short sequence motif ^306^N(G/D)W^308^ located in the β20–β21 loop. This exposed site is likely to be involved in interaction with Mfa1 and thus to be important for the function of BT1062. The spatial arrangement of the potential membrane-attachment site and protein–protein interaction site may be functionally significant as the location of the potential membrane-attachment site would allow more freedom and accessibility of a membrane-attached elongated molecule.

## 4. Conclusions

Bioinformatics studies, combined with experimental evidence from the related bacteria *P. gingivalis*, allow us to identify at least one putative operon that is likely to be involved in fimbrial assembly in *B. thetaiotaomicron* and other related bacteria. Our structural studies of the BT1062 protein in this operon revealed surprising structural similarities to the minor pilin GBS52 of *S. agalactiae* and the major pilin Spy0128 of *S. pyogenes*, both of which are Gram-positive bacteria. We demonstrated that a tandem repeat of the transthyretin-like fold is also likely to be adopted by other components of *Bacteroides* fimbriae, such as the major pili subunit FimA of *P. gingivalis*. These results may suggest a common evolutionary origin for this type of fimbrial component in both Gram-negative and Gram-positive bacteria. Thus, our studies contribute new insights into the evolution of fimbriae (pili).

Additional information about BT1062 is available from *TOPSAN* (Krishna *et al.*, 2010 ▶) at http://www.topsan.org/explore?pdbID=3gf8.

## Supplementary Material

PDB reference: BT1062, 3gf8

## Figures and Tables

**Table 1:** Summary of crystal parameters, data-collection and refinement statistics for BT1062 (PDB code 3gf8) Values in parentheses are for the highest resolution shell.

	λ_1_ MAD-Se	λ_2_ MAD-Se	λ_3_ MAD-Se
Space group	*P*4_1_2_1_2		
Unit-cell parameters (Å)	*a* = 106.9, *c* = 79.1		
Data collection			
Wavelength (Å)	0.9793	0.9116	0.9792
Resolution range (Å)	29.7–2.2 (2.26–2.20)	29.6–2.2 (2.26–2.20)	29.7–2.2 (2.26–2.20)
No. of observations	98494	94479	94046
No. of reflections	23880	23862	23868
Completeness (%)	99.9 (99.9)	99.9 (100)	99.9 (99.8)
Mean *I*/σ(*I*)	10.8 (1.8)	11.6 (2.2)	10.5 (1.6)
*R*_merge_ on *I*†	0.11 (0.72)	0.10 (0.65)	0.11 (0.80)
*R*_meas_ on *I*‡	0.13 (0.82)	0.11 (0.75)	0.13 (0.93)
Model and refinement statistics			
Resolution range (Å)	29.2–2.2		
No. of reflections (total)	23827		
No. of reflections (test)	1220		
Completeness (%)	99.8		
Data set used in refinement	λ_2_ MAD-Se		
Cutoff criterion	|*F*| > 0		
*R*_cryst_§	0.191		
*R*_free_¶	0.229		
Stereochemical parameters			
Restraints (r.m.s. observed)			
Bond lengths (Å)	0.016		
Bond angles (°)	1.52		
Average isotropic *B* value†† (Å^2^)	37.6		
ESU‡‡ based on *R*_free_ value (Å)	0.17		
Protein residues/atoms	284/2321		
Solvent molecules	175		

† *R*_merge_ = 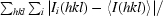 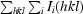.

‡ *R*_meas_ is the redundancy-independent *R*_merge_ (Diederichs & Karplus, 1997 ▶; Weiss & Hilgenfeld, 1997 ▶).

§ *R*_cryst_ = 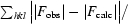 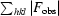, where *F*_calc_ and *F*_obs_ are the calculated and observed structure-factor amplitudes, respectively.

¶ *R*_free_ is the same as *R*_cryst_ but for 5% of the total reflections chosen at random and omitted from refinement.

†† This value represents the total *B* that includes TLS and residual *B* components.

‡‡ Estimated overall coordinate error (Collaborative Computational Project, Number 4, 1994 ▶; Cruickshank, 1999 ▶).
